# Dietary Phytochemicals in Health and Disease: Mechanisms, Clinical Evidence, and Applications—A Comprehensive Review

**DOI:** 10.1002/fsn3.70101

**Published:** 2025-03-19

**Authors:** Md. Sakhawot Hossain, Md Abdul Wazed, Sharmin Asha, Md. Ruhul Amin, Islam Md Shimul

**Affiliations:** ^1^ Department of Nutrition and Food Technology Jashore University of Science and Technology Bangladesh; ^2^ School of Nutrition and Public Health, College of Health Oregon State University Corvallis OR USA

**Keywords:** bioactive compounds, clinical evidence, dietary phytochemicals, health, therapeutic applications

## Abstract

Phytochemicals are bioactive compounds found in plants that play a key role in promoting health and preventing diseases. Present in fruits, vegetables, grains, and seed oils, these compounds are considered safe for consumption due to the co‐evolution and adaptation between mammals and plants. Due to their wide‐ranging biological effects, they have attracted considerable research interest. This comprehensive review explores the mechanisms of action, health benefits, and applications of dietary phytochemicals, with a particular focus on key groups such as polyphenols, flavonoids, and carotenoids. Research shows that dietary phytochemicals interact with nuclear and membrane receptors, influence metabolic pathways, and affect epigenetic modifications. Our review highlights the broad range of biological activities of these compounds, including antioxidant, antibacterial, anti‐inflammatory, anti‐diabetic, and anticancer effects, all of which contribute to their health‐promoting properties. Clinical evidence supports their role in the prevention and management of diseases such as cardiovascular disorders, metabolic conditions, and cancer, with diets rich in phytochemicals being linked to a lower risk of disease. Phytochemicals are also at the cutting edge of applications in food preservation, dietary supplements, and emerging medical treatments. Additionally, we identified advancements in extraction and identification techniques, particularly in metabolomics, which further enhance their applications in these areas. Despite their promising benefits, challenges such as bioavailability, regulatory barriers, and the need for robust clinical trials persist. However, innovative delivery systems like nanoparticles, liposomes, and encapsulation offer potential solutions to enhance bioavailability by improving absorption and stability. The review concludes by emphasizing the potential of personalized nutrition and combination therapies to enhance the health benefits of dietary phytochemicals while stressing the need for advancements in extraction methods, clinical trials, and bioavailability.

## Graphical abstract

This review highlights the health benefits and mechanisms of dietary phytochemicals, including their roles in disease prevention, antioxidant activity, and immune support. It also discusses their applications in the food and pharmaceutical industries.

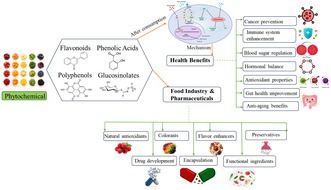

## 1 Introduction

Phytochemicals are naturally occurring compounds found in plants, which contribute to their color, flavor, and resistance to disease. Phytochemicals can be broadly grouped into a variety of classes, such as polyphenols, flavonoids, carotenoids, alkaloids, and glucosinolates, according to their different chemical structures and biological functions (Dagdag et al. 2024; Nigar et al. 2025). For example, flavonoids are one of the largest groups of polyphenols, largely present in fruits, vegetables, tea, and wine. They are known for their antioxidant properties and their ability to modulate cell signaling pathways (Rathee et al. 2024). On the other hand, carotenoids, including beta‐carotene and lycopene, are responsible for the red, orange, and yellow colors of some fruits and vegetables. They are recognized as providing support to the eyes and immune function (Rathee et al. 2024). Other important phytochemicals include glucosinolates, which are precursors to biologically active compounds found in cruciferous vegetables like broccoli and kale. These compounds are believed to have properties that may help prevent various types of cancer. Alkaloids, present in plants such as coffee and cocoa, act as central nervous system (CNS) stimulants (Alum and Ugwu 2023). Typically, these phytochemicals are consumed through a regular diet of fruits, vegetables, whole grains, nuts, and seeds. With their diverse properties, phytochemicals contribute to a wide range of health benefits, from reducing inflammation to preventing chronic diseases (Thacker and Ram 2024). The rising health awareness and preference for natural, plant‐based solutions have fueled the demand for phytochemical‐rich diets and functional foods. Global functional food sales reached $176 billion in 2013 (Baghdasaryan and Martirosyan 2024) and are projected to grow to $228.79 billion by 2025 (Subramanian and Anandharamakrishnan 2023), reflecting the shift toward natural alternatives to pharmaceuticals. Phytochemicals can play an important role in preventing mainly cardiovascular diseases and cancer. Through various mechanisms, phytochemicals combat oxidative stress (Ashraf et al. 2024), modulate gene expression (Khan et al. 2024), enhance cellular defenses (Gupta et al. 2023), and reduce inflammation (Guo, Li, et al. 2024). These actions collectively protect against chronic diseases, improve immune function, slow aging processes, and promote overall well‐being, underscoring their vital role in human health. Epidemiological studies consistently demonstrate that diets rich in phytochemicals lower the incidence of chronic diseases (Thacker and Ram 2024). For instance, an increased intake of phytochemical‐rich fruits and vegetables has been associated with a reduced risk of heart diseases and certain cancers (Pons 2024; Xu et al. 2024). Certain phytochemicals have been linked to better cardiovascular health and cancer prevention. This is likely due to their antioxidant properties (Cooperstone and Schwartz 2024) and their ability to affect metabolic pathways (Dagdag et al. 2024). They also play a role in DNA and cell damage repair, helping to maintain genomic stability and prevent mutations that can lead to cancer (Murai et al. 2024). Additionally, phytochemicals help regulate cholesterol levels (Paul et al. 2024), aid in weight management (Batool et al. 2025), promote cognitive function (Talib et al. 2024), physical health (Adeniran 2024), and immune response (Okafor et al. 2024).

Table 1 summarizes common dietary phytochemicals, highlighting their primary sources and associated bioactivities. These compounds, derived from plant‐based foods, are recognized for their potential health benefits, including antioxidant, anti‐inflammatory, and disease‐preventive properties. Despite extensive research on dietary phytochemicals, significant gaps remain in understanding their precise mechanisms of action and their long‐term effects on health. The variations in bioavailability and efficacy across different populations are also not well understood. This review critically evaluates mechanisms, clinical evidence, and applications of dietary phytochemicals in health and disease, synthesizing current research to advance our knowledge regarding their role in disease prevention and health promotion and highlighting important gaps in knowledge. As research on these bioactive compounds continues to grow, there is an increasing need to synthesize the existing evidence to better understand their role in promoting human health and preventing disease.

**TABLE 1:** Common dietary phytochemicals, their sources, and bioactivities.

Phytochemical category	Common phytochemicals	Sources	Primary bioactivities	References
Polyphenols 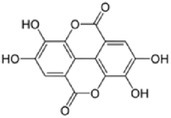	Beta‐carotene	Carrots, sweet potatoes, spinach	Antioxidant, vision health, immune system support	Cooperstone and Schwartz (2024)
	Lycopene	Tomatoes, watermelon, pink grapefruit	Antioxidant, prostate health, cardiovascular health	Fernandez‐Pan et al. (2024)
	Lutein	Kale, spinach, corn, egg yolk	Vision health, antioxidant	Eom et al. (2023)
Flavonoids 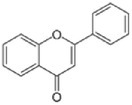	Quercetin	Apples, onions, berries	Antioxidant, anti‐inflammatory, cardiovascular health	Xu et al. (2024)
	Catechins	Green tea, cocoa, berries	Antioxidant, anti‐carcinogenic, weight management	Soboleva et al. (2023)
	Anthocyanins	Blueberries, blackberries, red cabbage	Antioxidant, anti‐inflammatory, cardiovascular health	Zhao, Wang, et al. (2024)
Phenolic acids 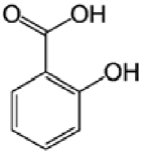	Caffeic acid	Coffee, berries, whole grains	Antioxidant, anti‐inflammatory, cardiovascular health	Bin‐Jumah et al. (2022)
	Ferulic acid	Oats, rice, eggplant, citrus fruits	Antioxidant, anti‐inflammatory, skin health	Mengli Zheng et al. (2024)
Glucosinolates 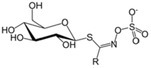	Sulforaphane	Broccoli, Brussels sprouts, cabbage	Detoxification, anti‐carcinogenic, antioxidant	Marshall et al. (2023)
	Indole‐3‐carbinol	Broccoli, kale, cabbage	Hormone regulation, detoxification, anti‐carcinogenic	Reyes‐Hernández et al. (2023)

## 2 Extraction and Identification of Phytochemicals

### 2.1 Traditional and Novel Extraction Methods

Phytochemical extraction has evolved from the traditional method to more advanced approaches, which have their advantages and limitations. Recent advances include methods like biphasic two‐stage deep eutectic solvent (TSDES), which combines hydrophilic and hydrophobic deep eutectic solvents with ultrasound‐assisted extraction for high yields and minimal solvent use (Dong et al. 2025), and natural deep eutectic solvent (NADES) systems, known for their high affinity for polyphenols and bioactives, offering a greener alternative to conventional methods (Iftikhar et al. 2025). Another emerging method, natural hydrophobic deep eutectic solvent (NaHDES), utilizes hydrophobic solvents from menthol and fatty acids, enhancing yield and sustainability (Sportiello et al. 2025). These modern approaches, while requiring careful optimization in terms of temperature, solvent‐to‐feed ratio, and processing time, promise high‐yield, eco‐friendly extraction methods that could revolutionize the sourcing of phytochemicals. In contrast, traditional methods of extraction, such as solvent extraction, steam distillation, and soxhlet extraction, have succeeded for many decades. Solvent extraction, using solvents like ethanol or methanol, is cheap and effective, but the process itself, however, needs large amounts of solvent and long processing times, therefore raising concerns about environmental sustainability (Liu et al. 2024). Steam distillation, the common method to extract essential oils, volatilizes compounds by steam passage; however, high temperatures can degrade heat‐sensitive phytochemicals (Kassie 2024). Soxhlet extraction is known for its high yield through continuous vaporization and condensation of the solvent; however, it shares environmental and time‐related drawbacks due to its high solvent consumption, which raises disposal and pollution concerns, and its lengthy extraction cycles, making the process time‐consuming (Jha and Sit 2022). To overcome these limitations, modern methods like supercritical fluid extraction (SFE), microwave‐assisted extraction (MAE), and ultrasound‐assisted extraction (UAE) have been developed. SFE, often using supercritical CO_2_, offers a high degree of extraction efficiency with very little solvent residue; however, it demands significant initial equipment costs (Usman et al. 2022). MAE improves efficiency, using microwaves to heat the solvent and plant material, which reduces extraction time and the quantity of solvents used, though it is probably not suitable for thermally sensitive compounds (Ferrara et al. 2023). The UAE uses ultrasonic waves to break down cell walls, helping release compounds in an eco‐friendly way (Sanjaya et al. 2022).

### 2.2 Techniques for Identifying Phytochemicals

Phytochemical identification relies on a combination of chromatographic and spectroscopic techniques, each with specific strengths for analyzing complex mixtures. Common chromatographic techniques, such as high‐performance liquid chromatography (HPLC), gas chromatography (GC), and thin‐layer chromatography (TLC), are normally used to isolate and identify phytochemicals by their chemical characteristics. HPLC has been effective in the separation of complex mixtures with a high resolution and was often a preliminary phase in phytochemical analysis (Parys et al. 2022). GC is very amenable to volatiles with excellent separation and quantification; however, the analytes need to be thermally stable (Trinklein et al. 2023). TLC is relatively less complex and inexpensive, although it provides less sensitivity and resolution compared to HPLC and GC; it is, therefore, suitable for preliminary compound identification (Radoičić et al. 2024). Spectroscopic methods, including mass spectrometry (MS), nuclear magnetic resonance (NMR), and infrared spectroscopy (IR), complement chromatographic techniques by clarifying molecular structures. MS has become an almost universal method for determining molecular weight and fragmentation patterns and is usually performed after HPLC or GC to provide compound separation (Kaczmarek et al. 2021). NMR is also very valuable for complex structural analysis in the identification of functional groups and molecular arrangements but requires larger sample sizes and a high degree of isolates purity (Kaczmarek et al. 2021). IR spectroscopy is very efficient for functional group identification, relying on vibrational energy absorption, and provides rapid identification with less detailed structural information than MS or NMR (Hong et al. 2021). Other emerging techniques include the use of metabolomics and chemometrics for phytochemical analysis. Metabolomics, the large‐scale study of small molecules, provides a profile of the phytochemical compositions and has been found to be particularly useful in the exploration of plant‐based medicines and nutritional compounds (Liang et al. 2024). Chemometrics combined with chromatographic and spectroscopic data enables better identification and improves the discrimination of complex phytochemical profiles among samples (Qin et al. 2024). Various methodologies for the extraction and identification of phytochemicals are summarized in Table 2. These methods highlight the diverse techniques employed to isolate bioactive compounds and their subsequent analysis.

**TABLE 2:** Extraction and identification methods for phytochemicals.

Method type	Technique	Description	Advantages	Limitations	Applications	References
Traditional extraction	Solvent extraction	Uses organic solvents to dissolve and extract phytochemicals	Cost‐effective, widely used	Low selectivity, solvent residue concerns	Extraction of flavonoids, alkaloids	Liu et al. (2024)
	Cold‐pressed oil extraction	Extraction from elderberry seeds using cold‐pressing method	High yield of essential fatty acids, tocopherols; minimal oxidation	Low oxidative stability, low emulsifiability	Nutritional oils, functional foods, antioxidant‐rich formulations	Siejak et al. (2025)
	Soxhlet extraction	Continuous extraction using boiling solvent	Efficient for solid samples	Time‐consuming, requires large solvent volumes	Essential oil extraction, polyphenol extraction	Kassie (2024); Liu et al. (2024)
Modern extraction	Biphasic temperature‐switchable deep eutectic solvent system (biphasic‐TSDES)	A system using hydrophilic and hydrophobic deep eutectic solvents combined with ultrasound‐assisted extraction	High extraction efficiency, selective, recyclable system	Requires optimization for different compounds, temperature dependency	Extraction of hydrophilic and hydrophobic phytochemicals from plant leaves, such as pigeon pea	Dong et al. (2025)
	Natural deep eutectic solvent (NADES) extraction	Utilizes a glycerol‐sodium acetate solvent system	Green solvent system, high affinity for polyphenols, better stability of bioactives	Requires precise optimization (shaking time, temperature, solvent‐to‐feed ratio), viscosity challenges	Extraction of polyphenols, flavonoids, antioxidants from fruits and seeds	Iftikhar et al. (2025)
	Natural hydrophobic deep eutectic solvent (NaHDES) extraction	Uses NaHDESs, made from menthol, fatty acids, and organic acids	Sustainable, high yield, environmentally friendly	Requires optimization, solvent‐to‐sample ratio sensitivity	Extraction of carotenoids from agro‐industrial by‐prod	Sportiello et al. (2025)
	Supercritical fluid extraction	Uses supercritical CO_2_ as a solvent under high pressure	High selectivity, minimal solvent residue	High initial setup cost, complex operation	Extraction of essential oils, bioactive compounds	Usman et al. (2022)
	Ultrasound‐assisted extraction	Applies ultrasonic waves to enhance solvent penetration	Reduces extraction time, energy‐efficient	Limited to small‐scale applications	Extraction of phenolic compounds, terpenoids	Riaz et al. (2024); Surmanidze et al. (2024)
Identification techniques	High‐performance liquid chromatography (HPLC)	Separates, identifies, and quantifies components in a sample	High resolution, suitable for nonvolatile compounds	Expensive equipment, requires skilled operation	Analysis of flavonoids, carotenoids	Parys et al. (2022)
	Gas chromatography–mass spectrometry (GC–MS)	Combines gas chromatography with mass spectrometry for identification	High sensitivity, suitable for volatile compounds	Not suitable for thermolabile compounds, high cost	Analysis of essential oils, fatty acids	Radoičić et al. (2024); Trinklein et al. (2023)
	Fourier transform infrared spectroscopy (FTIR)	Measures absorption of infrared light by chemical bonds	Fast, nondestructive analysis	Limited to functional group identification	Identification of functional groups in phytochemicals	Krishnan et al. (2023)

### 2.3 Examples of Commonly Studied Phytochemicals

Phytochemicals can be broadly classified into several categories, including polyphenols, flavonoids, carotenoids, and other bioactive compounds such as alkaloids, glucosinolates, and terpenes. Each group of phytochemicals possesses distinct biological activities linked with a wide variety of health benefits regarding antioxidant, anti‐inflammatory, and anticancer properties.

#### 2.3.1 Polyphenols

Polyphenols are a complex group of bioactive compounds, including flavonoids, phenolic acids, and tannins, which are widely distributed in fruits, vegetables, cereals, and various beverages like wine, tea, and coffee. Representative examples of polyphenolic compounds include resveratrol from grapes, oleuropein from olives, and epicatechins from tea and dark chocolate (Bin‐Jumah et al. 2022). The diversity of these compounds is well recognized for their high antioxidant activities and has been associated with a lower incidence of certain chronic diseases, such as cardiovascular disease, diabetes, and some cancers (Bin‐Jumah et al. 2022; Eom et al. 2023). The general extraction of polyphenols involves solvents like methanol, ethanol, or acetone; SPE is usually used to purify the extract after extraction for further analysis. Commonly, for qualitative and quantitative analysis, techniques included HPLC, LC–MS, and GC–MS (30–31). The technique of FTIR has also proved very useful in the analysis of polyphenolic compounds against a complex matrix of plant origin (Santos et al. 2024).

#### 2.3.2 Flavonoids

Flavonoids represent a broad class of polyphenolic compounds abundant in fruits, vegetables, and beverages, including tea and wine. Common dietary sources include citrus fruits, berries, apples, onions, and tea, while representative examples of flavonoids include quercetin, catechins, and anthocyanins (Xu et al. 2024). These compounds are highly valued for their antioxidant, anti‐inflammatory, and anticancer properties, and their dietary intake is associated with a reduced risk of cardiovascular diseases and certain cancers (Soboleva et al. 2023; Zhao, Wang, et al. 2024). Various techniques have been employed in the identification and quantification of flavonoids, which include HPLC with UV or mass spectrometric detection (Saad Rasheed et al. 2024). Additionally, commonly used methods for increasing yield and improving the efficiency of extraction include solid phase microextraction (SPME) and enzyme assisted extraction (EAE) (Agatonovic‐Kustrin et al. 2023).

#### 2.3.3 Carotenoids

Carotenoids are a group of naturally occurring pigments responsible for the yellow, orange, and red colors of many fruits and vegetables, including tomatoes, carrots, and leafy greens. Carotenoids also possess antioxidant properties, and their conversion to vitamin A is essential for maintaining proper vision and immune function (Cooperstone and Schwartz 2024). Beta‐carotene, lutein, and zeaxanthin are among the most widely studied carotenoids, with significant applications in promoting eye health and protecting against oxidative stress (Bin‐Jumah et al. 2022). Carotenoid extraction usually involves methods like HPLC for identification and quantification, with the selection of the solvent being an important factor in the process. For efficient carotenoid extraction, techniques like pressurized liquid extraction (PLE) and UAE are considered optimal (Agatonovic‐Kustrin et al. 2023). Other methods, such as spectrophotometry and capillary electrophoresis, are also used for carotenoid analysis (Popescu et al. 2022).

#### 2.3.4 Other Phytochemicals

Apart from polyphenols, flavonoids, carotenoids, and many other phytochemicals have drawn great interest due to their health‐promoting attributes. Alkaloids are nitrogenous compounds present in coffee, tea, and tobacco, among others. Known examples are caffeine, theobromine, and nicotine (Kamiloglu et al. 2022). These compounds show stimulant activities, and their therapeutic efficiencies are still under study in pain management and anti‐inflammatory, and anticancer therapies (Kamiloglu et al. 2022). Phase II detoxifying enzymes help eliminate harmful substances by attaching small molecules like glutathione to them, making them easier to excrete. Glucosinolates from cruciferous vegetables activate these enzymes, aiding in the detoxification of carcinogens and reducing cancer risk (Rizwan and Masoodi 2024). The analysis of glucosinolates in cruciferous plants commonly utilizes HPLC, along with other techniques such as LC–MS and enzyme‐linked immunosorbent assay (ELISA) (Abdelshafeek and El‐Shamy 2023). Terpenes, another class of bioactive phytochemicals, are found in essential oils of herbs such as mint, basil, and thyme. Terpenes are known for their antimicrobial, anti‐inflammatory, and anticancer properties; they are usually extracted through either steam distillation or cold pressing, while analysis is normally performed by GC coupled with MS (Siddiqui et al. 2024; Thurman 2020).

## 3 Bioactivities of Phytochemicals

### 3.1 Antioxidant Properties

Phytochemicals exhibit potent antioxidant properties by neutralizing free radicals and reactive oxygen species (ROS), which help prevent oxidative stress and cellular damage (Ashraf et al. 2024). Compounds like polyphenols, flavonoids, and carotenoids also play a key role in regulating antioxidant enzymes such as superoxide dismutase (SOD) and catalase, further safeguarding cells from oxidative damage (Rasmi et al. 2022). It has been documented that polyphenols such as quercetin and resveratrol from fruits, vegetables, and tea, and flavonoids from citrus fruits, berries, and onions exert antioxidant effects mainly through their free radical‐scavenging properties and the enhancement of antioxidant enzyme activity (Bin‐Jumah et al. 2022; Eom et al. 2023). Meanwhile, antioxidant carotenoids, like beta‐carotene and lutein, protect tissues, especially the eyes, from oxidative stress. Beta‐carotene is found in carrots, while lutein is found in leafy vegetables (Xu et al. 2024). The antioxidant activity of these phytochemicals has significant health implications, including the prevention of chronic diseases like cardiovascular disease (Cooperstone and Schwartz 2024), neurodegenerative disorders (Ayaz et al. 2024), and aging (Talib et al. 2024). Diets rich in antioxidants, particularly polyphenols and flavonoids, have been associated with a reduced risk of heart disease (Muscolo et al. 2024), while compounds like resveratrol have shown protective effects in neurodegenerative diseases such as Alzheimer's (Ayaz et al. 2024). Furthermore, antioxidants help to slow the aging process by mitigating oxidative damage to skin and muscle tissues (Muscolo et al. 2024). Overall, the antioxidant properties of phytochemicals contribute to long‐term health by reducing oxidative damage and preventing chronic diseases.

### 3.2 Antibacterial Properties

Some phytochemicals possess high antibacterial activity by disrupting bacterial cell membranes, interfering with bacterial enzymes, and interfering with the replication of bacterial DNA. These mechanisms help fight against bacterial infections, especially antibiotic‐resistant bacteria. Phytochemicals may interfere with bacterial cytoplasmic membranes by causing leakage of cellular contents or inhibit an important enzyme involved in cell wall synthesis or energy production. For example, allicin, produced from garlic, effectively exerts an antibacterial effect against Gram‐positive and Gram‐negative bacteria by invading bacterial enzymes, thus compromising cell membrane integrity (Okafor et al. 2024). In addition, curcumin from turmeric demonstrates antibacterial activity by targeting bacterial DNA and inhibiting enzymes involved in bacterial metabolism (Murai et al. 2024). Tannins, found in tea, exhibit antibacterial properties by binding to bacterial cell walls, preventing bacterial adhesion and growth. These compounds have proved to be highly effective against pathogens such as *Escherichia coli* and *Staphylococcus aureus* (Villanueva et al. 2023). They can find potential use in medicine and food preservation due to the antibacterial property of phytochemicals. In medicine, they could be used as an alternative or complementary therapy to antibiotics, especially against multi‐drug‐resistant bacteria, with ongoing clinical trials about the efficacy of compounds like curcumin and allicin (Murai et al. 2024; Okafor et al. 2024). In food preservation, phytochemicals such as tannins and allicin can help extend shelf life by preventing microbial spoilage and improving food safety, offering a natural alternative to synthetic preservatives (Rudrapal et al. 2023; Villanueva et al. 2023).

### 3.3 Anticancer Properties of Phytochemicals

Phytochemicals have potential anticancer activities through mechanisms involving the induction of apoptosis, inhibition of cell proliferation, and prevention of angiogenesis (Batool et al. 2025; Varadharajaperumal et al. 2024). These compounds interfere with the signaling pathways of cancerous cells and thus are useful in cancer research and therapy. For instance, curcumin and epigallocatechin gallate induce the apoptosis of cancer cells by altering mitochondrial membrane potential, releasing cytochrome c, and activating caspases. They also suppress anti‐apoptotic proteins such as Bcl‐2 and increase the levels of pro‐apoptotic molecules like Bax, which further facilitate the death of cancer cells (Batool et al. 2025). Besides, phytochemicals such as genistein may arrest unchecked cancer cell proliferation via the regulation of cyclins and CDKs. More precisely, genistein causes G2/M phase arrest in breast cancer cells through the downregulation of CDK1 and cyclin B1 (Varadharajaperumal et al. 2024). Angiogenesis is the process of forming new blood vessels, which plays a role in tumor growth and spread. Phytochemicals like resveratrol and curcumin can block this process. These compounds work by influencing angiogenic factors such as vascular endothelial growth factor (VEGF) and its receptors. Specifically, curcumin reduces the number of blood vessels in tumors, leading to a lack of nutrients and oxygen for tumor cells (Kumari et al. 2024). Phytochemicals also disrupt key cancer signaling pathways; for instance, epigallocatechin gallate (:EGCG) inhibits epidermal growth factor receptor (EGFR) signaling, reducing cell proliferation and promoting apoptosis, while curcumin targets the NF‐κB pathway, suppressing inflammatory cytokines and survival genes (Ullah et al. 2024). Table 3 provides a comprehensive overview of the bioactivities associated with key phytochemicals. These include antioxidant, anti‐inflammatory, antimicrobial, and anticancer properties, among others.

**TABLE 3:** Summary of bioactivities of key phytochemicals.

Phytochemical	Source	Bioactivity	Reported effects	References
Curcumin	Turmeric ( *Curcuma longa* )	Antioxidant, anti‐inflammatory, anticancer	Reduces oxidative stress, inhibits tumor growth	Murai et al. (2024); Okafor et al. (2024)
Catechins	Green tea ( *Camellia sinensis* )	Antioxidant, cardioprotective	Lowers blood pressure, improves heart health	Soboleva et al. (2023)
Resveratrol	Grapes, red wine	Antioxidant, anti‐aging, neuroprotective	Protects neurons, reduces risk of age‐related diseases	Ayaz et al. (2024); Bin‐Jumah et al. (2022); Eom et al. (2023)
Quercetin	Onions, apples	Antibacterial, anti‐inflammatory	Inhibits bacterial growth, reduces inflammation	Xu et al. (2024)
Lycopene	Tomatoes	Antioxidant, anticancer	Protects against prostate cancer, reduces cholesterol	Cui et al. (2024); Miszczyk et al. (2024)
Allicin	Garlic ( *Allium sativum* )	Antibacterial, antifungal, cardioprotective	Lowers blood pressure, combats infections	Okafor et al. (2024)
Epigallocatechin gallate (EGCG)	Green tea	Antioxidant, anticancer	Suppresses tumor cell proliferation, prevents DNA damage	Adeniran (2024); Chen et al. (2024); Talib et al. (2024)
Anthocyanins	Berries (e.g., blueberries)	Antioxidant, anti‐inflammatory	Reduces risk of cardiovascular diseases, improves vision	Batool et al. (2025); Ye et al. (2024)

While phytochemicals in whole foods are generally considered safe, clinical trials have found potential adverse effects linked to isolated phytochemical supplements. For instance, high doses of green tea catechins have been associated with liver toxicity in some people, prompting regulatory warnings about excessive consumption (Acosta et al. 2023). Similarly, too much intake of isoflavones from soy has been linked to hormonal imbalances, especially in postmenopausal women (Soukup et al. 2023). These findings highlight the need for caution when applying the health benefits of phytochemicals from observational studies to clinical settings. More rigorous long‐term trials are necessary to determine safe and effective dosage ranges, particularly for phytochemical supplements.

#### 3.3.1 Human Studies

Human studies have shown how different phytochemicals have great potential in cancer prevention and treatment. A study conducted in the United States reported that curcumin has powerful anticancer action against colon cancer, inducing programmed cell death and hence enhancing the prognosis. Poor bioavailability of curcumin is enhanced by the use of nanoparticulate systems, and its potential as a treatment has been validated in clinical trials (Li 2023). Similarly, EGCG, a major catechin in green tea, has exhibited remarkable anticancer properties by inhibiting the proliferation and metastasis of breast and prostate cancers. It modulates key signaling pathways such as MAPK and PI3K/AKT, showing anti‐proliferative, anti‐angiogenic, and pro‐apoptotic actions across various tumor models (Talib et al. 2024). Genistein is a soy isoflavone acting as a chemopreventive agent against hormone‐dependent tumors. It inhibits tyrosine kinases, diminishes angiogenesis, and triggers apoptosis in prostate and breast cancer models. Clinical trials indicate that genistein decreases prostate‐specific antigen (PSA) levels in patients with prostate cancer (Van der Eecken et al. 2023). Another promising compound for chemoprevention is curcumin, which enhances the efficacy of conventional therapies. In pancreatic cancer, the combination of curcumin with gemcitabine enhances therapeutic efficacy and impairs chemoresistance by targeting multiple signaling pathways (Kothawade et al. 2024; Sharma et al. 2024).

### 3.4 Bioactive Properties of Phytochemicals in Health Management

Among these phytochemicals, most importantly gingerol has demonstrated very significant anti‐inflammatory properties, which include the inhibition of pro‐inflammatory cytokines, a reduction in NF‐κB activation, and the alleviation of oxidative stress. Compounds found in ginger, especially 6‐gingerol, suppress the production of inflammatory mediators and cytokines, thereby reducing inflammation. It is also confirmed in studies that gingerol reduces the immunogenicity of dendritic cells, further contributing to its anti‐inflammatory effects (Pázmándi et al. 2024). Omega‐3 fatty acids, especially those of flaxseed origin, have also been shown to have anti‐inflammatory effects through the inhibition of NF‐κB activation and the regulation of inflammatory cytokine expression, which decreases oxidative stress (Kumar Maurya 2024). Phytochemicals such as berberine and anthocyanins are recently being researched for their diabetic management. Berberine, a compound extracted from various plants, was identified to activate AMPK, which increases insulin sensitivity and cellular glucose uptake (Yakin et al. 2024). Similarly, anthocyanins, abundant in berries, improve insulin resistance and glucose metabolism to regulate blood sugar levels and reduce complications in diabetic individuals (Ye et al. 2024). Phytochemicals exhibit a wide variety of other bioactivities, including anti‐obesity, neuroprotective, and cardioprotective effects. Compounds such as resveratrol and curcumin inhibit adipogenesis and therefore help with weight management and reduce the risk of obesity (Batool et al. 2025; Kumari et al. 2024). Phytochemicals like curcumin and EGCG protect against neurodegenerative diseases by modulating oxidative stress and inflammatory pathways, showing promise in delaying neurodegeneration and supporting cognitive function, particularly in aging populations (Talib et al. 2024). Phytochemicals like omega‐3 fatty acids contribute to cardiovascular health by improving lipid profiles, reducing blood pressure, and lowering the risk of cardiovascular diseases (Kumar Maurya 2024). However, it is important to consider the potential adverse effects of high‐dose phytochemical supplementation. For example, while polyphenols are generally beneficial, they can exhibit pro‐oxidant properties at high concentrations, potentially leading to cellular damage instead of protection (Dzah et al. 2024). Similarly, curcumin, which is widely studied for its anti‐inflammatory properties, may cause gastrointestinal discomfort and liver toxicity when consumed in excessive amounts (Chrubasik‐Hausmann 2023). This emphasizes the need for appropriate control of phytochemical intake so that their advantages are maximized without raising the risk of harmful side effects. An integrated approach is required to explore the therapeutic value of phytochemicals with due consideration for their possible risks.

## 4 Mechanisms of Action of Phytochemicals

These bioactive compounds exert their effects through various mechanisms, including interactions with cellular pathways, enzymes, and receptors.

### 4.1 Interaction With Nuclear Receptors

It can bind to nuclear receptors, such as estrogen receptors, peroxisome proliferator‐activated receptors (PPARs), and liver X receptors (LXRs), thereby modulating gene expression that influences various physiological processes, like metabolism and cellular differentiation.

#### 4.1.1 Phytoestrogens and Estrogen Receptors

**FIGURE 1:**
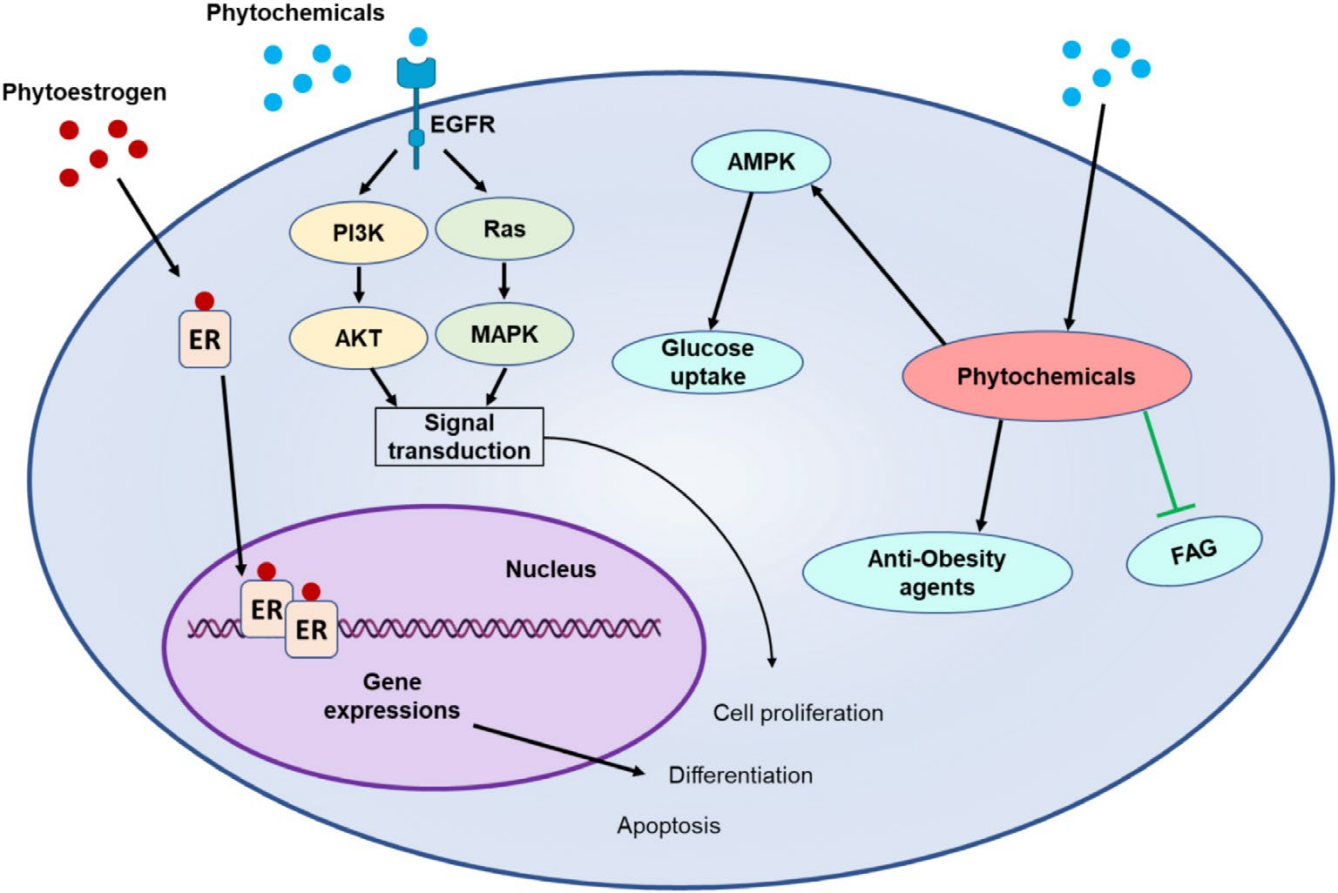
Mechanisms of action of phytochemicals: Receptor interactions and metabolic modulation.

Phytoestrogens are plant‐derived compounds that mimic or modulate estrogen activity in the body by binding to estrogen receptors. These compounds, found in foods like soy and flaxseed, interact with estrogen receptors in a way similar to the body's natural estrogen, potentially influencing various physiological processes, including hormonal balance and disease prevention. Phytoestrogens interact with estrogen receptors (ERs), influencing various biological processes, such as hormone regulation, bone health, and cancer prevention. These phytoestrogens act as agonists or antagonists to mimic endogenous estrogen by binding to ERs, thus activating estrogen‐responsive genes while also, in particular contexts, competing with endogenous estrogen for its proliferative action (Ariyani et al. 2023; Elsayed et al. 2022). This dual action allows phytoestrogens to influence important biological processes such as cell proliferation, differentiation, and apoptosis. For instance, the interaction of genistein with the ERs has been associated with health benefits in hormone‐dependent conditions such as breast cancer. Phytoestrogens are said to minimize the risk of cancer through their role in modulation of estrogenic action, thereby overcoming the proliferative action of endogenous estrogen in the breast (Talib et al. 2024; Van der Eecken et al. 2023). Other than anticancer properties, phytoestrogens are also researched for mitigation of menopausal symptoms. Their mild estrogenic effects have been shown to reduce hot flashes, improve bone density, and enhance overall quality of life, making them a promising natural alternative to hormone replacement therapy (Shikh and Makhova 2023). However, the effects of phytoestrogens vary across tissues and are influenced by factors such as dosage, tissue type, and an individual's hormonal status. Phytoestrogens can have protective effects, such as in the breast. However, high doses or use in individuals with high estrogen levels may increase estrogen activity in tissues like the uterus, potentially raising the risk of diseases like endometrial cancer (Szukiewicz 2023). Figure 1 illustrates the diverse mechanisms through which phytochemicals exert their bioactivities. These mechanisms include direct interactions with cellular receptors, modulation of signaling pathways, and alterations in metabolic processes.

### 4.2 Interaction With Membrane Receptors

#### 4.2.1 Tyrosine Kinase Inhibitors and EGFR

Some phytochemicals have natural tyrosine kinase inhibitor activity, targeting membrane receptors such as the epidermal growth factor receptor. The latter plays a critical role in cell proliferation, survival, and metastasis. Activation of EGFR subsequently initiates downstream signaling cascades involving PI3K/AKT and MAPK pathways that often are overexpressed in cancers (Kaveh Zenjanab et al. 2024). Phytochemicals inhibit these oncogenic cascades through direct inhibition of EGFR signaling, thus impairing tumor development and inducing programmed cell death in tumor cells (Adeniran 2024). Quercetin, a flavonoid found in fruits, vegetables, and grains, is a prominent example of a phytochemical that inhibits EGFR signaling. It suppresses EGFR‐mediated activation of the PI3K/AKT and MAPK pathways, reducing cancer cell proliferation and inducing apoptosis in lung, breast, and colon cancer models (Kaveh Zenjanab et al. 2024). Similarly, EGCG, the primary polyphenol in green tea, inhibits EGFR signaling by reducing PI3K/AKT and MAPK pathway activation. This action leads to decreased cell survival and enhanced apoptosis, particularly in breast and prostate cancers (Talib et al. 2024). Quercetin and EGCG show promise as complementary cancer therapies by inhibiting EGFR signaling and modulating related pathways, which enhance the effectiveness of conventional EGFR‐targeting treatments like tyrosine kinase inhibitors and cetuximab. These phytochemicals may also help tackle treatment resistance, improving therapeutic outcomes (Damare et al. 2024). However, further clinical studies are necessary to establish their safety, optimal dosage, and long‐term efficacy as an adjuvant in cancer therapy.

### 4.3 Modulation of Metabolic Pathways

A promising approach to treating metabolic disorders is through phytochemicals, which have the capacity to influence key metabolic pathways. The process is driven by the activation of AMP‐activated protein kinase (AMPK), a key regulator of cellular energy balance. AMPK activation enhances glucose uptake and fatty acid oxidation while inhibiting lipogenesis, thereby promoting improved energy balance (Zhao, Yan, et al. 2024). Indeed, phytochemicals like astaxanthin, a marine carotenoid, exert protective effects on insulin signaling and glucose metabolism, leading to improved glucose and lipid homeostasis (Medoro et al. 2024). Moreover, phytochemicals, such as tea polyphenols, ellagic acid, and curcumin, become anti‐obesity agents through epigenetic mechanisms. These phytochemical compounds suppress the activities of important enzymes participating in lipogenesis, such as FAS—a key enzyme involved in the process of adipogenesis and fat accumulation (Chen et al. 2024; Worthmann et al. 2024). Phytochemicals from Korean traditional foods, including flavonoids and saponins, also demonstrate anti‐obesogenic properties through the regulation of lipid metabolism and gut microbiota composition (Das et al. 2024). More importantly, a few phytochemicals increase sensitivity to insulin and enhance glucose metabolism by affecting glucose transporters and, hence, are used in treating metabolic disorders such as obesity and diabetes, and cardiovascular diseases (Paul et al. 2024; Shuvo et al. 2023). Berberine, an alkaloid contained in plants of the genus Berberis, is one of the most important phytochemicals that could represent metabolic advantages. Berberine activates AMPK, improving insulin sensitivity and enhancing glucose uptake. Clinical studies have shown that berberine supplementation significantly reduces blood glucose levels and improves lipid profiles in individuals with type 2 diabetes (Zhang et al. 2024). Another noteworthy compound is EGCG, a catechin from green tea. EGCG inhibits FAS, reducing lipogenesis and promoting fat oxidation, thereby aiding in weight management and lowering the risk of cardiovascular diseases (Adeniran 2024; Chen et al. 2024).

### 4.4 Epigenetic Modifications

Phytochemicals have drawn a wide range of interest due to their potential activity of bringing about epigenetic modifications that change the expression of genes without any alteration in the DNA sequence. These include DNA methylation, histone modification, and regulation of noncoding RNAs; all of these are essential in determining gene activity (Gu et al. 2024). A notable example is sulforaphane from cruciferous vegetables such as broccoli, which has been known to demethylate tumor suppressor genes. By reactivating these silenced genes, sulforaphane promotes anti‐cancer activity, offering a preventive and therapeutic approach to cancer management (Sailo et al. 2024). Similarly, curcumin, a key bioactive compound in turmeric, influences histone acetylation, a process that enhances gene expression while suppressing oncogenes. This dual action contributes significantly to curcumin's anti‐cancer properties by restoring normal cellular functions and inhibiting tumor progression (Khan et al. 2024). This epigenetic modulation by phytochemicals has particular significance in cancer prevention and treatment, providing a new therapeutic approach resulting from the reactivation of tumor suppressor genes and silencing oncogenes. The therapeutic potential of these compounds extends beyond cancer treatment, offering a way to overcome resistance to conventional therapies and improve clinical outcomes. Table 4 outlines the various mechanisms through which phytochemicals influence biological systems. Phytochemicals show promise in targeting and reversing epigenetic changes, paving the way for advanced prevention and integrated therapeutic strategies (Sharma et al. 2024).

**TABLE 4:** Mechanisms of action of phytochemicals.

Phytochemical	Biological target	Mechanism of action	Effects	References
Curcumin	NF‐κB (nuclear factor kappa B)	Inhibits NF‐κB activation, reducing pro‐inflammatory cytokines	Anti‐inflammatory, reduces chronic inflammation	Ullah et al. (2024)
Resveratrol	SIRT1 (sirtuin 1), AMPK (AMP‐activated protein kinase)	Activates SIRT1 and AMPK pathways, enhancing mitochondrial function	Anti‐aging, improves metabolic health	Rogina and Tissenbaum (2024)
Quercetin	PI3K/Akt pathway, MAPK pathway	Modulates cell signaling pathways, inducing apoptosis in cancer cells	Anticancer, reduces tumor progression	Adeniran (2024); Talib et al. (2024)
Lycopene	IGF‐1R (insulin‐like growth factor 1 receptor)	Inhibits IGF‐1R signaling, slowing down cancer cell proliferation	Anticancer, prevents prostate cancer	Tjahjodjati et al. (2020)
EGCG	EGFR (epidermal growth factor receptor), DNA	Binds to EGFR, inhibiting cell proliferation and DNA methylation	Anticancer, prevents tumor growth	Chen et al. (2024); Damare et al. (2024)
Anthocyanins	Nrf2 (nuclear factor erythroid 2–related factor 2)	Activates Nrf2, upregulating antioxidant defense enzymes	Antioxidant, protects against oxidative damage	Dong et al. (2024)
Allicin	Membrane receptors, enzymes	Inhibits enzyme activity (e.g., acetyl‐CoA synthetase), affecting bacterial membranes	Antibacterial, disrupts bacterial cell function	Bhattacharya et al. (2022)
Genistein	Estrogen receptors, tyrosine kinases	Binds to estrogen receptors, inhibits tyrosine kinases	Anticancer, prevents hormone‐related cancers	Gupta et al. (2023)

It is important to understand how phytochemicals work for their use in therapy. Recent studies suggest that plant polyphenols may impact aging and immune function (Ayaz et al. 2024; Murai et al. 2024); however, their exact roles in the body are still unclear. Since whole foods contain many phytochemicals, it is hard to identify which ones are active and how they interact. More research is needed to fully understand these effects.

## 5 Health Benefits of Phytochemicals

### 5.1 Prevention and Treatment of Chronic Diseases

#### 5.1.1 Cardiovascular Diseases

Phytochemicals have gained significant attention for their potential cardioprotective effects in preventing and treating cardiovascular diseases (CVD). These compounds contribute to cardiovascular health through various mechanisms, including reducing oxidative stress, improving lipid profiles, lowering blood pressure, and preventing low‐density lipoprotein (LDL) oxidation, all of which are crucial in the pathogenesis of CVD (Aguayo‐Morales et al. 2024). Oxidative stress, another critical component of endothelial dysfunction and atherosclerosis, is clearly diminished by the antioxidant actions of phytochemicals that neutralize free radicals and prevent cellular injury (Arandhara et al. 2024). Many phytochemicals, such as flavonoids (e.g., quercetin) and polyphenols (e.g., resveratrol), have vasodilatory effects. They improve blood vessel function and lower blood pressure by boosting nitric oxide production (Harahap et al. 2024). These compounds also affect lipid metabolism by reducing LDL cholesterol levels and increasing high‐density lipoprotein HDL cholesterol, thereby reducing atherosclerosis and associated complications such as heart attack and stroke (Eilam et al. 2022). Further, the prevention of LDL oxidation by phytochemicals is an important factor in the prevention of atherosclerotic plaque formation. Notable examples of cardioprotective phytochemicals include quercetin, which is shown to lower blood pressure and improve endothelial function, and resveratrol, which activates sirtuins and reduces oxidative stress, enhancing cardiovascular protection (Eilam et al. 2022; Kumari et al. 2024).

#### 5.1.2 Metabolic Disorders

Recent meta‐analyses have examined the influence of various polyphenols and flavonoids on cardiovascular health and metabolic syndrome (MetS). The intake of total flavonoids, flavan‐3‐ols, isoflavones, stilbenes, flavones, and quercetin was inversely associated with the risk of MetS (Ramaiah et al. 2024). Quercetin supplementation, specifically, significantly decreased fasting blood glucose and systolic blood pressure SBP (Noshadi et al. 2024). In addition, compounds like docosahexaenoic acid, eicosatetraenoic acid, inorganic nitrates, and resveratrol may help reduce blood pressure in older adults. However, these findings are limited by biases and need further confirmation (Kujawska et al. 2024).

#### 5.1.3 Cancer Prevention and Therapy

Recent studies have highlighted the potential of dietary polyphenols and aromatic herbs in the prevention and management of metabolic disorders as well as in supporting cancer prevention and therapy. Polyphenols have been associated with significant cardiometabolic improvements in key markets, including weight, BMI, and CRP level reductions, with the highest category intake of flavonoids, flavan‐3‐ols, isoflavones, stilbenes, flavones, and quercetin associated with a reduced risk of metabolic syndrome (Chew et al. 2024; Ramaiah et al. 2024). Specifically, supplementation with cinnamon (≤ 2 g/day) improves glycemic control, lipid profile, and BMI in patients with type 2 diabetes (Garza et al. 2024). Another study reported positive effects of spices including black cumin, ginger, turmeric, and saffron, which improve fasting glucose levels; black cumin and ginger also tend to lower levels of glycated hemoglobin (HbA1c; Garza et al. 2024). In the setting of cancer, phytochemicals remain of therapeutic interest. An umbrella meta‐analysis highlighted that dietary carotenoids could reduce the risk of several cancers. However, it also noted that high‐dose carotenoid supplements, typically exceeding 15–20 mg per day, may increase cancer risk in some instances, particularly when taken over extended periods (Sui et al. 2024). Curcumin, a well‐researched natural compound, has demonstrated effectiveness in animal studies, especially for glioma therapy. Glioma refers to a type of brain tumor that arises from glial cells, which support and protect neurons (Luís et al. 2024). Lycopene, found in tomatoes, has been associated with a reduced risk of prostate cancer, although the evidence remains inconclusive (Cui et al. 2024). Moreover, metastasis‐directed therapy is emerging as one of the promising ways of managing oligometastatic prostate cancer, thus delaying the use of androgen‐deprivation therapy (Miszczyk et al. 2024).

### 5.2 Impact on Mental Health and Cognitive Function

Phytochemicals have demonstrated considerable potential in improving mental health and cognitive function through inhibition of chronic neuroinflammation and oxidative stress, the major causes of age‐related cognitive decline and mental health disorders such as depression and anxiety. Accordingly, these compounds modulate inflammatory pathways, scavenge free radicals, and offer support to neural cell integrity, thus promoting brain health (Wang et al. 2024). For example, *Curcuma longa* bioactive product from curcumin showed its efficacy in traumatic brain injury models through mechanisms of reducing oxidative stress, reducing inflammatory cytokines, and cerebral edema improvement (Guo, Li, et al. 2024). Phytochemical‐rich dietary patterns, such as the Mediterranean diet and calorie restriction, have been associated with positive cognitive outcomes. Indeed, a meta‐analysis of such diets in randomized controlled trials demonstrated that they have significant benefits on global cognitive function, speed of performance, and executive function in healthy adults (Guo, Tian, et al. 2024). Polyphenol supplementation, particularly flavonoids at a daily dose of ≥ 579 mg, has been shown to enhance immediate memory retrieval in overweight individuals over 60 years old, emphasizing its potential role in age‐related cognitive health (Farag et al. 2024).

### 5.3 Immune System Enhancement

Phytochemicals stimulate immune function through the activation of immune cells, prevention of chronic inflammation, and gut health, which are all important in maintaining healthy immunity. These compounds can also stimulate T cells and macrophages into action, enabling the body to fight against infection and control inflammation more effectively. Additionally, phytochemicals promote healthy gut microbiota, which are crucial for immune regulation, and their anti‐inflammatory properties help to prevent the excessive inflammation linked to chronic diseases and immune dysfunction (Lew and Fleming 2024). For example, epigallocatechin‐3‐gallate (EGCG) exhibits anti‐inflammatory effects and shows potential in treating inflammatory bowel disease, with an effective dosage range of 32–62 mg/kg/day (Wei et al. 2024). Specific phytochemicals, like echinacea, beta‐glucans, and catechins, further illustrate their immune‐boosting properties. Echinacea has been shown to activate macrophages and natural killer cells, contributing to the prevention of upper respiratory infections (Shao et al. 2024). Beta‐glucans from oats and mushrooms have also been shown to enhance immune responses by activating immune cells, hence reducing the risk of infections and managing chronic inflammation (Van Doan et al. 2024). Various catechins found in green tea show anti‐inflammatory activity and also enhance the functional ability of immune cells toward infection prevention and recovery processes (de Oliveira Assis et al. 2024). These are highly beneficial to vulnerable people, like the elderly or those who have compromised immune systems, since enhancing immune responses can make significant improvements in health outcomes in conditions such as chemotherapy or autoimmune diseases (Traidl‐Hoffmann 2024). While phytochemical‐containing diets have been linked with reduced disease risk by research, the associations do not always imply causation. For example, while observational studies suggest that high intake of polyphenols will reduce cardiovascular disease incidence, clinical trials often report inconsistent results due to confounding factors like diet patterns and genetics (Ebrahimi et al. 2023; Rudzińska et al. 2023). Also, phytochemical effects may vary among populations; a meta‐analysis indicated that resveratrol significantly improved metabolic diseases in diabetic or obese subjects, but healthy individuals responded minimally (Kujawska et al. 2024). These findings emphasize the importance of personalized nutrition strategies rather than assuming that phytochemicals benefit everyone equally.

## 6 Epidemiological and Clinical Evidence

### 6.1 Summary of Epidemiological Studies

Recent epidemiological studies consistently show that diets rich in antioxidants and polyphenols are associated with a reduced risk of chronic diseases, such as cardiovascular disease, diabetes, and certain types of cancer. A meta‐analysis suggests that higher intakes of vitamins C, E, and β‐carotene are linked to a reduced risk of type 2 diabetes, likely through enhanced insulin sensitivity and mitigation of oxidative stress (Lampousi et al. 2024). Another meta‐analysis reported that higher consumption of different types of polyphenols, especially flavonoids, was significantly associated with 22% lower odds of metabolic syndrome (Ramaiah et al. 2024). Diets rich in polyphenol‐containing plant‐based foods are associated with a 7% reduction in all‐cause mortality, as evidenced by findings from large cohort studies (Zupo et al. 2024).

### 6.2 Clinical Trials and Their Findings

Clinical trials are most important in validating the health benefits of phytochemicals, providing controlled evidence of their efficacy, safety, and mechanisms to prevent or treat diseases. Recent studies have explained the therapeutic potentials of phytochemicals such as curcumin and resveratrol in several disease conditions. Curcumin supplementation significantly lowered alanine aminotransferase (ALT) and aspartate aminotransferase (AST) levels in nonalcoholic fatty liver disease (NAFLD), with pooled mean differences of −5.61 and −3.90 units/L, respectively, indicating improved liver function (Lukkunaprasit et al. 2023). Curcumin doses ranging from 50 to 3000 mg per day, taken for 8–12 weeks, have been found effective for managing this condition (Ebrahimzadeh et al. 2024; Vajdi et al. 2024). Curcumin is also a promising drug in traumatic brain injury models, as evidenced by the attenuation of inflammatory cytokines, cerebral edema, and neuroprotective factor upregulation (Guo, Li, et al. 2024). Resveratrol supplementation has been shown to reduce testosterone, luteinizing hormone, and dehydroepiandrosterone sulfate levels in patients with polycystic ovary syndrome, potentially enhancing ovarian function (Fadlalmola et al. 2023). Animal models of ischemic stroke have shown resveratrol reduces infarct size, edema, and blood–brain barrier impairment, highlighting its neuroprotective properties (López‐Morales et al. 2023). Despite promising findings, clinical trials often encounter challenges, such as variability in bioavailability, dosing regimens, and participant heterogeneity, all of which can influence outcomes. For example, the low bioavailability of curcumin limits its therapeutic potential in certain populations. However, advancements such as nanoparticle‐based formulations have shown promise in overcoming this barrier, potentially enhancing its efficacy (Khosravi and Seifert 2024). There is also much variability in formulations, like whole foods versus extracts, and participant lifestyle factors such as diet, exercise, and concurrent medication use that greatly affect the study results. Moreover, the effectiveness of phytochemicals can also vary among populations and trial designs; several studies have shown no significant benefits, particularly in small sample sizes or short treatment periods (Rudzińska et al. 2023). Resveratrol has shown inconsistent outcomes, with some trials failing to find significant improvements in metabolic health or cardiovascular function, raising questions about its clinical significance (Godos et al. 2024). Limitations in trial design, dosing regimens, and inconsistent outcome measures highlight the need for standardized approaches to advance the understanding and application of phytochemicals (Guo, Li, et al. 2024). Table 5 summarizes recent epidemiological and clinical studies on phytochemicals, providing insights into their health benefits and therapeutic potential.

**TABLE 5:** Epidemiological and clinical findings on phytochemicals.

Phytochemical	Study details	Findings	Disease risk reduction	Relevance	Reference
Curcumin	5‐week animal study with OLETF rats (T2DM model)	Improved glucose homeostasis, lipid profile, cognitive function, and reduced inflammation and ER stress with exercise + curcumin	Potential to mitigate diabetes‐related cognitive dysfunction	Suggests combined diet and exercise benefits in T2DM	Cho et al. (2020)
Resveratrol	Phase 2 RCT with 100 COVID‐19 outpatients in Ohio	Lower rates of hospitalization, ER visits, and pneumonia vs. placebo	Potential reduction in severe COVID‐19 complications	Highlights resveratrol's antiviral and anti‐inflammatory potential in COVID‐19 treatment	McCreary et al. (2022)
Quercetin	8‐week study on streptozotocin induced diabetic rat models	Restored body weight, insulin, glucose, and renal parameters; reduced inflammation and oxidative stress; improved kidney histology	Potential prevention of diabetes‐associated kidney dysfunction	Highlights quercetin's reno‐protective and antidiabetic effects	Rahmani et al. (2023)
Lycopene	Review of preclinical prostate cancer models	Demonstrated anti‐cancer activity in xenograft, chemically‐induced, and transgenic mouse models; outcomes depend on dose and timing	Potential to reduce prostate cancer risk	Highlight's role of lycopene and tomatoes in prostate cancer prevention	Moran et al. (2022)
EGCG	2‐month RCT with 50 T2DM patients	Improved antioxidant capacity, lipid profile, blood pressure, and AIP	Reduced cardiovascular risk and oxidative stress	Highlights EGCG's potential benefits in T2DM management	Bazyar et al. (2021)
Anthocyanins	24‐week RCT with 206 older adults (MCI or cardiometabolic disorders)	No significant group difference in episodic memory, but improved memory slope in weeks 8–24	Potential to slow cognitive decline	Suggests anthocyanins may aid in dementia prevention	Aarsland et al. (2023)
Allicin	4‐week study on ethanol‐fed mice	Reduced gut permeability, altered microbiota, and suppressed hepatic inflammation via CD14‐TLR4 pathway	Potential to mitigate alcohol‐induced liver inflammation	Highlights gut‐liver axis modulation by allicin	Panyod et al. (2020)
Soy Isoflavones	48‐month prospective study with 1460 BC survivors	Moderate pre‐ and post‐diagnosis intake reduced all‐cause and BC‐specific mortality (up to 66% lower risk)	Improved survival outcomes in BC survivors	Suggests soy's safety and benefits in BC prognosis	Ho et al. (2021)
Enterolactone	5‐year study with 2105 BC survivors	Improved short‐term survival; other phytoestrogens linked to poorer prognosis	Potential BC survival benefit	Highlights enterolactone's short‐term effects	Jaskulski et al. (2020)

### 6.3 Correlation Between Dietary Intake and Disease Risk Reduction

Phytochemicals have a relationship with reducing the risk of noncommunicable diseases and generally improving health. A diet rich in fruits, vegetables, whole grains, nuts, and seeds is the primary source of phytochemicals and has constantly been related to lower risks of cardiovascular diseases, cancer, diabetes, and neurodegenerative disorders. For example, berries and cherries are rich in flavonoids and polyphenols, which exert antioxidant, anti‐inflammatory, and vasodilatory actions that enhance endothelial function, lower blood pressure, and improve lipid profiles, in turn contributing to cardiovascular health (Carvalho et al. 2024). Population studies also reveal the inverse relationship of dietary phytochemical intake to the incidence of colorectal, breast, and prostate cancers (Bin‐Jumah et al. 2022; Kaveh Zenjanab et al. 2024; Kumari et al. 2024; Talib et al. 2024). Moreover, anthocyanins in dark‐colored fruits such as blueberries enhance insulin sensitivity and glycemic control, while phytochemicals such as curcumin from turmeric and catechins from green tea exert neuroprotective actions, minimizing the development of Alzheimer's and Parkinson's disease (Kim et al. 2024; Ye et al. 2024). Public health guidelines emphasize the protective benefits of plant‐based diets, with recommendations such as “five‐a‐day” for fruits and vegetables and adherence to diets like the Mediterranean and DASH diets, which are rich in phytochemical‐containing foods. Studies suggest plant‐based diets can reduce coronary artery disease risk by up to 29% compared to nonvegetarian diets (Mehta et al. 2023). The dose–response relationship is important because the cardiovascular benefits of flavonoids seem to level off at moderate daily doses, around 500 mg/day. Taking more than this amount doesn't provide additional benefits (Micek et al. 2021). Similarly, a randomized trial demonstrated that 100 mg/day of soy isoflavone supplements decreased mammographic density, a biomarker of breast cancer risk, among Asian women around the time of menopause (Rajaram et al. 2023). Notably, synergy of whole foods often results in significantly greater magnitude of effects compared to isolated compounds alone; for instance, whole apples have greater antioxidant effects than equivalent doses of the isolated compound quercetin (Xia et al. 2024). These findings have influenced national public health policies worldwide because national dietary guidelines in countries such as the U.S. and Canada now emphasize phytochemical‐rich food groups such as whole grains and legumes, which are also recognized for their role in combating NCDs and promoting health (Cara et al. 2023; Luongo et al. 2024; Viroli et al. 2023). Epidemiological studies have reported associations between phytochemical‐dense diets and decreased risk of some diseases. However, these observational studies have limitations, such as confounding factors and dependence on self‐reported dietary information, which can lead to bias. Additionally, while these associations are interesting, they don't prove that phytochemicals directly cause health benefits. To make clear recommendations, randomized controlled trials are needed to confirm these relationships and identify the best dosages and forms of phytochemicals for therapy.

## 7 Applications of Phytochemicals

Phytochemicals are utilized across various sectors due to their bioactive properties and health benefits. In health and nutrition, they are key components of functional foods, nutraceuticals, and dietary supplements, promoting overall well‐being and reducing the risk of chronic diseases.

### 7.1 Use in Food Preservation and Processing

#### 7.1.1 Natural Preservatives

Phytochemicals are increasingly recognized as natural alternatives to synthetic preservatives due to their antimicrobial, antioxidant, and anti‐enzymatic properties. Protection of food by these compounds is achieved by preventing the growth of spoilage organisms and pathogenic organisms through disruption of cell membranes, interference with metabolic pathways, and scavenging free radicals. For example, phenolic compounds, including flavonoids and tannins, through their antimicrobial activity, attack bacterial cell walls and proteins, while their antioxidant activity prevents lipid oxidation, which is one of the main reasons for rancidity and deterioration in the quality of food (Pons 2024; Xu et al. 2024). Certain phytochemicals, such as catechins and chlorogenic acid, inhibit enzymatic browning in fruits and vegetables, enhancing their stability and visual appeal during storage. Notable examples include rosemary extract, which contains carnosic acid and carnosol—potent antioxidants effective in preventing lipid oxidation in meat and oil‐based products (Andrade et al. 2022; Campolina et al. 2023). Essential oils from oregano, thyme, and clove, rich in compounds like thymol, carvacrol, and eugenol, demonstrate strong antimicrobial activity against both Gram‐positive and Gram‐negative bacteria, making them effective for extending the shelf life of perishable foods, particularly meats (Ricardo‐Rodrigues et al. 2024). Similarly, EGCG tends to retard the growth of bacteria and check lipid peroxidation, thereby acting as a preservative in dairy and meat products (Talib et al. 2024).

#### 7.1.2 Applications in the Food Industry

In general, phytochemicals are used within the food industry as natural preservatives and functional components in order to enhance the safety, shelf life, and quality of foods from many different product categories. Green tea extract improves the shelf life and functional properties of yogurt and cheese (Celik et al. 2023), while essential oils and plant extracts protect bread and cookies from fungal contamination and lipid oxidation (Konfo et al. 2023). Phytochemical‐fortified edible coatings, such as chitosan films with incorporated oregano oil, are used on fruits and vegetables to control microbial deterioration and extend the storage life of these commodities (Cruz‐Monterrosa et al. 2023). Biodegradable films containing essential oils, such as oregano, have been shown to exhibit antimicrobial activity against *Escherichia coli* and *Staphylococcus aureus* by reducing microbial loads in fresh produce (Abd‐Alhadi et al. 2023; Mouhoub et al. 2023). Incorporating green tea extract into biodegradable packaging enhances antioxidant activity, extending the shelf life of fatty foods like packaged meat (Andrade et al. 2022). Cinnamon essential oil incorporated into coatings on fruits, for example, strawberries, reduces microbial growth, maintains nutritional quality, and improves sensory appearance (Piechowiak and Skóra 2023). Meanwhile, grape seed extract‐based starch films are antimicrobial and antioxidant active, along with high mechanical strength, UV protection, and high‐water resistance, which are rather suitable for preserving high‐moisture‐containing foods (Meixia Zheng et al. 2023). Furthermore, curcumin‐based biopolymer coatings exhibit excellent antimicrobial activity against *Listeria monocytogenes* and *Salmonella spp*., making them highly effective in preserving the safety and quality of perishable foods (Sánchez‐Bodón et al. 2024).

### 7.2 Potential in Biomedical Fields

**FIGURE 2:**
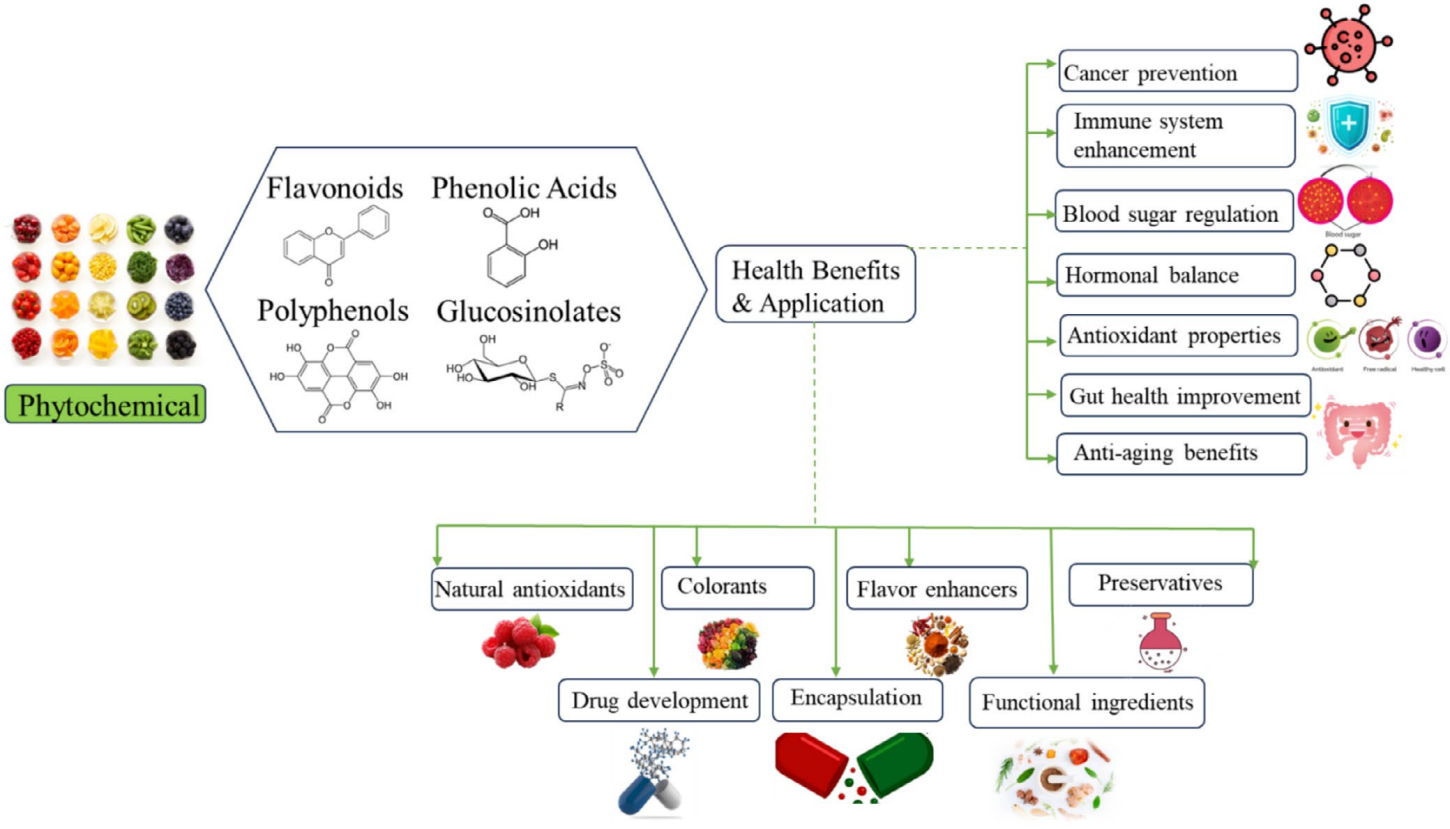
Health benefits and applications of phytochemicals.

Phytochemicals are very important in the development of supplementary diets that are available as capsules, tablets, powders, and liquid extracts. The conditions to consider when formulating these drugs include bioavailability, stability, and convenience to the consumer. Liquid extracts provide fast absorption, whereas the capsule and tablet forms ensure controlled dosages and are easy to store for later use (Kato et al. 2023). Challenges like poor solubility and stability are being addressed through innovations such as encapsulation, liposomal delivery systems, and nanoparticle‐based formulations, all of which enhance the bioavailability of phytochemicals (Aatif 2023). The increasing consumer demand for clean‐label products, driven by greater awareness, has led to growth in the market for plant‐based dietary supplements (Tachie et al. 2023). The demand increased more during the COVID‐19 pandemic when people looked toward supplements that boost and protect immunity (Djaoudene et al. 2023). New formulations employing plant by‐products as natural additives in processed foods were innovations that have arisen from such an industry. Some of the challenges include nutritional adequacy, problems in safety, and consumer acceptance issues, among others (Tachie et al. 2023). Despite the many impediments to making it a mainstream product, interest in sustainable and health‐promoting products remains strong, driving the continued growth of the plant‐based supplement market. Figure 2 illustrates the diverse health benefits and applications of phytochemicals.

## 8 Future Directions and Research Gaps

### 8.1 Areas Needing Further Research

The potential of phytochemicals is often limited by challenges such as low bioavailability, rapid metabolism, and poorly understood mechanisms of action, hindering their full therapeutic efficacy. Due to poor absorption and rapid metabolism, a number of them, including polyphenols and carotenoids, reach target sites in very low levels (Aatif 2023; Meixia Zheng et al. 2023). Future research should investigate how factors like gut microbiota composition, genetic polymorphisms, and co‐administration with other compounds influence the bioavailability of phytochemicals. To address these challenges, innovative delivery systems such as nanoparticles, liposomes, and encapsulation show promise in enhancing absorption and stability, thereby unlocking their full therapeutic potential. Additionally, while phytochemicals like curcumin and resveratrol are known to modulate inflammatory and oxidative stress pathways, their precise interactions with cellular signaling, transcription factors, and epigenetic processes require further investigation (Ayaz et al. 2024; Bin‐Jumah et al. 2022; Murai et al. 2024; Okafor et al. 2024).

### 8.2 Potential for Novel Therapeutic Applications

Phytochemicals, with their diverse bioactive properties, offer significant potential for novel therapeutic strategies, particularly in personalized medicine, emerging diseases, and combination therapies. Phytochemicals like polyphenols, isothiocyanates, and curcumin can modulate the gut microbiota, influencing various health outcomes. Polyphenols promote beneficial bacteria such as *Bifidobacterium* and *Lactobacillus*, which help improve metabolic health and reduce inflammation in conditions like obesity and diabetes (Albin et al. 2024). Similarly, isothiocyanates found in cruciferous vegetables support gut microbes and reduce cancer risk by modulating gut inflammation (Na et al. 2023). Additionally, curcumin impacts the gut‐brain axis, helping to reduce neuroinflammation, which could offer potential benefits for neurodegenerative diseases like Alzheimer's (Kim et al. 2024). In personalized medicine, phytochemicals can be customized to individuals' genetic, environmental, and lifestyle factors, optimizing their efficacy. For example, genetic variations in enzymes like cytochrome P450 and GST influence the metabolism and bioactivity of compounds such as isothiocyanates, which are linked to cancer prevention (Mazari et al. 2023; Sharma et al. 2024). Phytochemicals also find their applications in emerging therapeutic areas, since their anti‐inflammatory and immunomodulatory properties show prospects for the treatment of long COVID‐19, autoimmune disorders, and neurodegenerative diseases like Alzheimer's and Parkinson's (Ayaz et al. 2024; Kaveh Zenjanab et al. 2024; Murai et al. 2024). In addition, phytochemicals combined with conventional treatments increase the therapeutic efficacy. For instance, curcumin and EGCG synergize with chemotherapy in enhancing cancer cell apoptosis while protecting normal cells; resveratrol enhances statin efficacy in the cardiovascular diseases process; and berberine improves glycemic control with antidiabetic drugs like metformin (Harahap et al. 2024; Rasmi et al. 2022; Talib et al. 2024; Ullah et al. 2024; Yakin et al. 2024).

### 8.3 Challenges in Phytochemical Research

Phytochemicals hold immense potential for improving health; however, they face challenges that limit their application in mainstream therapeutics and dietary supplements. Studies on bioactive compounds show that they often have low bioavailability due to their poor solubility and stability. Their effectiveness can be reduced as they are affected by processes like digestion, absorption, and metabolism after ingestion (Hu et al. 2023). Similarly, a clinical study found that oral resveratrol results in low bioavailability (Szymkowiak et al. 2025), and an animal model shows the same for phytochemicals like quercetin. However, the IQC‐γCD inclusion complex increased bioavailability 3.8‐fold in sprague–dawley rats and 10.5‐fold in healthy humans (Kapoor et al. 2021). Many phytochemicals degrade when exposed to environmental factors like pH changes, heat, light, oxygen, and prooxidants, reducing their concentration in foods at consumption. However, recent strategies, such as structural changes, colloidal systems, and nanotechnology, have been introduced to improve the absorption and bioavailability of bioactive compounds (Vieira and Conte‐Junior 2024).

Another critical issue is the lack of standardization and quality control in cultivation, extraction, and storage, leading to variability in product composition and efficacy, such as inconsistent polyphenol levels in green tea supplements (Badmanaban et al. 2020). Regulatory hurdles add complexity, as organizations like the U.S. Food and Drug Administration (FDA) and the European Food Safety Authority (EFSA) require robust evidence of safety and efficacy, which can be challenging to provide due to the diverse mechanisms of action of phytochemical mixtures (Santini et al. 2018). Moreover, funding limitations restrict essential large‐scale clinical trials and the development of advanced delivery systems (Fareed and Ali 2022). Addressing these challenges through standardized protocols, regulatory reforms, and enhanced funding will be vital for advancing phytochemical research and applications. Additionally, exploring phytochemicals as adjuvants in immunotherapy or antimicrobial resistance could expand their therapeutic applications.

### 8.4 Future Directions in Phytochemical Research

To advance the field of phytochemical research, future studies should prioritize several key areas. First, standardization of study protocols is necessary to provide consistent methodologies, which will allow for easier comparison of findings and increase the validity of results. Examining gut microbiota interactions is also important to determine how individual microbiomes affect phytochemical metabolism and bioactivity, as differences in gut flora can result in varying health effects. Because the majority of phytochemicals possess poor bioavailability, novel delivery systems must be explored. Long‐term clinical trials must be conducted to ascertain the extended effects and safety of phytochemicals across various populations. Mechanistic studies that examine the molecular targets upon which phytochemicals act will define their therapeutic promise and limitations. Bioavailability studies need to be targeted toward the revelation of different forms of phytochemicals' metabolism and absorption so that they can provide information for proper dietary guidance and supplement formulation. Instead of using general population trials, tailored trials in high‐risk groups such as those with insulin resistance or neurodegenerative diseases would prove to be more significant. While phytochemicals are considered to be safe, systematic assessments of potential adverse effects in large‐scale trials are essential to establish safe upper intake levels. Research should also examine the application of individualized nutrition models, based on genetic and metabolic profiles, to enhance dietary interventions. Furthermore, governments should fund research involving the inclusion of phytochemicals in school and hospital feeding programs to assess long‐term health effects. Cost–benefit studies of phytochemical‐containing diets compared to pharmacological treatments can inform healthcare policy. Lastly, phytochemical research is faced with regulatory challenges due to variation in extraction methods, dosing, and regulation. Therefore, the establishment of international quality control standards for purity and potency, harmonization of supplement labeling policies, and study of food‐matrix effects on absorption are imperative. By filling these gaps, future studies can enable more definitive evidence of the role of phytochemicals in health and disease prevention and their safe and effective use in dietary recommendations and therapeutic applications.

## 9 Conclusions

Phytochemicals have immense potential to enhance health and the prevention of chronic diseases through their antioxidant, anti‐inflammatory, and metabolic regulatory properties. However, their efficacy is often hindered by low bioavailability and potential adverse effects when consumed in high doses, particularly in isolated supplements. As research progresses, it is crucial to standardize methodologies and conduct rigorous long‐term studies to establish safe dosage ranges and assess the health implications of phytochemicals across diverse populations. Apart from that, phytochemicals have proven to be powerful natural preservatives, highlighting their valuable function in the food sector. Understanding the interaction between phytochemicals and gut microbiota could unveil new therapeutic applications and lead to personalized nutrition strategies founded on individual metabolic profiling. Through the inclusion of phytochemicals in functional foods and dietary interventions, we are able to realize their health effects in a secure and efficient way. Finally, addressing regulatory challenges and promoting evidence‐based applications of phytochemicals will enhance their role in public health. This holistic approach not only supports disease prevention and health promotion but also aligns with global health initiatives aimed at sustainable, natural solutions for improving overall well‐being.

## Author Contributions

**Md. Sakhawot Hossain:** conceptualization (lead), data curation (lead), investigation (lead), methodology (lead), resources (lead), software (lead), validation (lead), visualization (lead), writing – original draft (lead). **Md Abdul Wazed:** conceptualization (equal), supervision (equal), visualization (equal), writing – review and editing (equal). **Sharmin Asha:** writing – original draft (equal), writing – review and editing (equal). **Md. Ruhul Amin:** visualization (supporting), writing – review and editing (equal). **Islam Md Shimul:** conceptualization (equal), supervision (equal), visualization (equal), writing – review and editing (equal).

## Ethics Statement

The authors have nothing to report.

## Consent

The authors have nothing to report.

## Conflicts of Interest

The authors declare no conflicts of interest.

## Data Availability

The data supporting this study's findings are available from the corresponding author upon request.
